# 1072. Red blood cell transfusion in acute cerebral injuries: a systematic review of preclinical animal studies

**DOI:** 10.1186/2197-425X-2-S1-P88

**Published:** 2014-09-26

**Authors:** M Laflamme, A Boutin, H Haghbayan, M Shemilt, F Lauzier, L Moore, R Zarychanski, J Lacroix, F Lamontagne, DA Fergusson, P Desjardins, AF Turgeon

**Affiliations:** Université Laval, Québec, Canada; CHU de Québec Research Center, Québec, Canada; University of Manitoba, Winnipeg, Canada; Université de Montréal, Montréal, Canada; Université de Sherbrooke, Sherbrooke, Canada; Ottawa Hospital Research Institute, Ottawa, Canada

## Introduction

In brain-injured patients, the appropriateness of restrictive red blood cell (RBC) transfusion strategies is uncertain given the brain's vulnerability to hypoxemia. Current practice is based on small clinical trials and physiologic data from clinical and preclinical studies. Little is known regarding how preclinical data has informed RBC transfusion in this specific patient population.

## Objectives

To evaluate the association between RBC transfusion strategies and important outcomes in animal models of acute cerebral injuries.

## Methods

We conducted a systematic review of studies evaluating RBC transfusion strategies in animal models of acute cerebral injuries. We searched MEDLINE, EMBASE and BIOSIS for preclinical comparative studies (randomized or non randomized studies) evaluating at least two different RBC transfusion strategies, including presence/absence of transfusion. We collected data on model characteristics, baseline sample characteristics, intervention, co-interventions and outcomes. Our primary outcomes were mortality and neurological function. Risk of bias was evaluated using a tool based on recommendations from the CAMARADES group [1]. Descriptive statistics using data reported in the original publications are presented.

## Results

We identified 6031 records and included 24 unique studies. Cerebrovascular injury models were used in 15 studies: stroke models (n=12), subarachnoid haemorrhage models (n=3). Traumatic brain injury models were used in 9 studies (trauma n=6, cryogenic lesion n=2, epidural lesion n=1). Twelve studies were performed using murine models. Most studies compared RBC transfusion to no transfusion (n=23) and used whole blood (n=21). Five studies reported data on mortality and 6 on neurological function; others reported surrogate endpoints such as cerebral blood flow. RBC transfusion was associated with decreased mortality in 3 of 5 (60%) studies reporting this outcome. In studies assessing neurological function, 2 of 6 studies favoured transfusion compared to no transfusion. No studies compared different transfusion thresholds. Hemodynamic and vital signs were not systematically reported in all studies. High risk of bias was observed in the majority (15/24, 58%) of studies (all studies reporting mortality and neurological function), notably lack of randomization and blinding of outcome assessment.

## Conclusion

Data from animal studies are insufficient to inform transfusion practice in patients with acute cerebral injuries. The use of standardized, controlled and validated animal models, clinically relevant transfusion strategies and longer follow-up periods may help future research in the field.
